# SER-CAT Studies: Implications to Dose Reduction by MDS (Multiple-DataSet) Data Collection Strategy

**DOI:** 10.1063/4.0001163

**Published:** 2025-10-27

**Authors:** Unmesh Chinte, Zheng-Qing (Albert) Fu, Zhongmin Jin, John Rose, John Chrzas, Bi-Cheng Wang

**Affiliations:** SER-CAT and University of Georgia, Athens, GA, USA

## Abstract

The Multiple-DataSet (MDS)1 data collection strategy was examined to determine if the method could be used to produce SAD data sets capable of phasing the structure with a lower X-ray dose. The tests were carried out using data collected on zinc-free Insulin crystals at SER-CAT 22ID beamline. In each experiment, a standard data set was collected followed by the collection of N low-exposure data sets collected using 1/Nth exposures to keep the total radiation dose the same in all cases.The anomalous signal was analyzed and a comparison was made between the standard data set and the merged low-exposure data set by MDS. The study showed that the anomalous signal (as defined by the Ras index2 and peak heights of sulfurs from Bijvoet difference Fourier maps) for the merged low-exposure (MDS) data set was significantly higher compared to the signal observed for the standard data set.

The analysis was then repeated merging fewer low-exposure data sets (e.g. N-1, N-2) and the anomalous signal in these cases was found to be comparable or higher than that observed for the standard set. This implies that, by using the MDS strategy, a crystal can be exposed to a lower radiation dose and can still provide sufficient anomalous signal for phasing. Thus, the MDS strategy may offer a better data collection option for radiation sensitive crystals and crystals with a lower symmetry, since it may provide enough data to solve the structure before significant radiation decay occurs. This may be more significant considering the highly intense beams currently available at synchrotron sources such as APS (Advanced Photon Source) after the recent upgrade.
